# High expression of the ADC target Claudin-6 associates with aggressive endometrial cancer and remains high in metastatic lesions

**DOI:** 10.1038/s44276-026-00225-x

**Published:** 2026-04-15

**Authors:** Vilde Linnea Gullovsen, Daniela Pavlicenco, Marta E. Hjelmeland, Kathrine Woie, Ingfrid S. Haldorsen, Jone Trovik, Hege F. Berg, Camilla Krakstad

**Affiliations:** 1 Centre for Cancer Biomarkers, Department of Clinical Science, University of Bergen, Bergen, Norway; 2 Department of Gynecology and Obstetrics, Haukeland University Hospital, Bergen, Norway; 3 Department of Clinical Medicine, University of Bergen, Bergen, Norway; 4 Mohn Medical Imaging and Visualization Centre, Department of Radiology, Haukeland University Hospital, Bergen, Norway

## Abstract

**Background:**

Patients with advanced or recurrent endometrial cancer (EC) have limited treatment options. The development of antibody-drug conjugates (ADCs) offers new treatment strategies by targeted delivery of cytotoxic payloads, including conventional chemotherapy, to cancer cells. An ADC targeting Claudin-6 is currently in clinical testing however expression patterns of Claudin-6 has not been thoroughly explored in EC.

**Methods:**

Membrane expression of Claudin-6 was evaluated by immunohistochemistry in a large, prospectively collected EC cohort with 1106 primary tumours and 187 metastatic lesions. Claudin-6 expression was scored according to the Gastric criteria and correlated with clinicopathological characteristics and follow-up data.

**Results:**

Claudin-6 expression was detected in 18% of ECs and was highly expressed in 10% of all samples. High expression was observed in 49% of serous tumours and 22% of carcinosarcomas. High Claudin-6 independently predicted poor disease-specific survival in multivariate analysis (HR: 1.7, CI: 1.2-2.4, *p* = 0.002). In the paired primary-metastasis cohort, Claudin-6 was highly expressed in 19% of primary tumours and, of these, 77% maintained high expression in at least one corresponding metastatic lesion.

**Conclusions:**

Our study identified Claudin-6 as an independent prognostic marker in EC and as a potential target for ADCs, particularly in serous and carcinosarcoma subtypes.

## Background

Endometrial cancer (EC) is the most common gynaecological malignancy in high-development index countries and has a 5-year overall survival rate of 84% [1]. Unfortunately, both the incidence and mortality rates of EC are rising [2]. Primary treatment for all risk categories is surgical removal of the uterus with or without adnexa and ovaries, often accompanied by sentinel lymph node dissection. Early-stage EC has a favourable prognosis, however, survival rates drop significantly for late-stage or recurrent disease. For patients classified as high risk, adjuvant chemotherapy is most often carboplatin combined with paclitaxel [3, 4] and recently immunotherapy was approved as an option for patients with advanced or recurrent tumouody-drug conjugates (ADCs) have emerged as promising new treatment options, binding drugs to antigen-specific antibodies to provide targeted therapies. An optimal ADC binds to specific antigens present on tumour cells thereby minimizing damage to healthy tissues [5]. Several ADCs are in ongoing clinical trials for EC, including ADCs targeting human epidermal growth factor receptor 2 (HER2), folate receptor alpha (FRα), trophoblast cell surface antigen 2 (Trop-2), nectin cell adhesion molecule 4 (NECTIN4), and B7 homolog four protein (B7-H4) [6, 7]. To improve the usefulness of ADCs, exploring expression of antigens may pinpoint which ADC that are most relevant for specific patient subgroups. In this context, the present study aimed to explore and evaluate the expression of Claudin-6 in EC.

To date, three different ADCs that target Claudin-6 are being evaluated in phase 1 clinical trials (NCT05103683, NCT05394675, NCT06932094), of which two trials have posted preliminary response data. The ADC TORL-1-23 is conjugated to a potent microtubule inhibitor Monomethyl Auristatin E (MMAE) and is being evaluated in patients with advanced solid tumours, including ECs. Preliminary data show that the TORL-1-23 is well tolerated with anti-tumour effects reported for CLDN6-positive platinum resistant ovarian cancers, with an objective response rate (ORR) of 67% (4/6) at medium dose (2.4 mg/kg). Response data from EC patients are not published yet. The second trial evaluates the Claudin-6 ADC DS-9606a, which has pyrrolobenzodiazepine (PBD) as payload. A favourable safety profile is reported with a confirmed objective response in preliminary analyses, awaiting more detailed response data. Preliminary data from these phase I trials are promising and demonstrate a potential of Claudin-6 as an ADC target.

Claudins are tight junction proteins important for cellular permeability, cell polarity, tissue integrity and signal transductions [8, 9]. Claudin-6 expression associates with favourable outcomes in breast cancer, non-small cell lung cancer and the CMS2-subtype of colorectal cancer [10–12]. Claudins are tight junction proteins important for cellular permeability, cell polarity, tissue integrity and signal transductions [8, 9]. Claudin-6 expression associates with favourable outcomes in breast cancer, non-small cell lung cancer and the CMS2-subtype of colorectal cancer [10–12]. However, Claudin-6 expression has also been linked to tumour invasion, increased proliferation and decreased apoptosis, as well as poor prognosis in bladder, cervical, endometrial and adrenocortical cancer [13–15]. Thus, the role of Claudin-6 may vary across tumour types, dependent on the environmental and molecular context. Claudin-6 shows limited expression in normal adult tissues and, with a link to aggressiveness in specific tumour types, Claudin-6 may act both as a prognostic marker and as a target for ADC-directed treatment [16].

In the present study, we evaluate Claudin-6 protein expression in a large cohort of EC patients including tissue from metastatic lesions, to pinpoint if specific subgroups of ECs may be particularly relevant for treatment with ADCs directed at Claudin-6.

## Methods

### Patient series

All included patients gave written informed consent. Tumour tissue from patients with endometrial cancer was prospectively collected and stored in the Bergen Gynaecological Cancer Biobank (REK 2014/1907). Clinicopathological data was retrieved from medical records and stored in Bergen Gynaecological Cancer Health Registry (approved by the Norwegian Data Inspectorate 2016/7421 and the Regional Ethical Committee REK 7226). A study specific approval was granted from the local ethical board to retrieve clinical information from the health registry and tumour samples from the biobank from patients included in this study (REK 2018/594). The population-based study cohort included samples collected between 2001 to 2021 (*n* = 1106). The number of samples are sufficient to detect a 20% difference in 5-year disease-specific survival (DSS) at a 5% significance level. Clinicopathological data included age, 2009 FIGO stage, histological type, tumour grade, tumour size, depth of myometrial infiltration, and follow-up information (mean follow up 72 months). Molecular classification was performed as previously described [17]. Postoperative risk groups was defined as low, intermediate and high according to ESMO guidelines [18].

### Immunohistochemistry (IHC)

Tumour tissue from 1106 endometrial cancer patients was collected on tissue microarrays (TMA) as previously described [19]. Briefly, full sections from formalin fixed paraffin embedded (FFPE) samples of primary tumours and metastatic lesions were prepared and stained with hematoxylin and eosin (HE). The tumour areas with the highest tumour content were identified on each slide. Three tissue cylinders (0.6 mm) were extracted from primary tumours FFPE slides, extracted from the donor blocks and embedded in recipient TMA blocks using a custom-made precision instrument (Beecher Instruments, Silver Spring, MD, USA). The TMA slides were prepared (5 µm slices) and one HE section was used for validation by a pathologist. For detection of Claudin-6, sections were deparaffinized in xylene and rehydrated in graded alcohol, before antigen retrieval by microwave boiling for 15 min (pH 9, S2367, Agilent Dako, Santa Clara, Ca, USA). Endogenous peroxidase activity was inhibited using peroxidase blocking solution (S2023, Dako, Denmark) for 8 min. The slides were incubated for 60 min with anti-Claudin-6 primary antibody (EPR28103-113, Abcam, Cambridge, UK) diluted 1:200, followed by 30 min incubation with anti-rabbit secondary antibody (K4003, Dako, Denmark). Diaminobenzidine peroxidase (DAB) (K3468, Dako, Denmark) was subsequently applied for 6 min at room temperature, and the slides were counterstained with hematoxylin (S3301, Dako, Denmark). The slides were dehydrated in graded alcohol, washed in xylene and mounted using Eukitt mounting medium.

### Evaluation of staining

Protein expression was evaluated according to the scoring guidelines for interpretation of HER2 IHC in gastric carcinoma [20]. Individual scores were based on the percentage of tumour cells exhibiting membranous staining and the intensity of the staining across the three cores. Only membrane staining was assessed, and cores containing insufficient tumour tissue were excluded from the evaluation. For metastases, one 1 mm core was available per lesion. A score of 0 was assigned when less than 10% of the tumour cells had membranous reactivity. If membranous reactivity was observed in more than 10% of the tumour cells, faint staining was defined as 1 + , complete, basolateral, or lateral staining with weak to moderate intensity was defined as 2 + , while 3+ required strong membranous reactivity in more than 10% of the tumour cells [21]. A kappa value was calculated to determine the consistency of the assessments. Two reviewers, VLG and DP independently scored random selections of TMA slides representing 110 patients, yielding a linear weighted kappa of 0.81 across all scorings. All scorings were performed blinded for clinicopathological characteristics and outcome.

### RNA sequencing

RNA was extracted from tumour samples using the AllPrep DNA/RNA mini kit (QIAGEN Nordic, Hvidovre, DK), according to the manufacturer’s protocol. Illumina TruSeq Total GOLD (illumina, San Diego, Ca, USA) was used to generate cDNA libraries (350 ng RNA input). Sequencing was conducted by Illumina HiSeq 4000 (paired end, 75 bp) and hiscat 2.0.5 was used to align raw reads to human genome GRCh38 with Gencode v26 used as transcriptome reference. Samtools and Feature Counts were used to process aligned files and to count the aligned reads, respectively. Normalization of counts and differential expression analyses were performed in DESeq2.

### Statistical analysis

Statistical evaluations were performed with IBM SPSS Statistics version 29.0.2.0 (IBM, NY, USA) [20]. Kaplan-Meier analysis was used to generate disease-specific survival (DSS) curves. The time of surgery was used as the starting point, with time to death due to endometrial cancer as the endpoint. Differences in survival between groups were assessed using the log-rank (Mantel-Cox) test. Categorical variables were analysed using Pearson Chi-square test or Fisher’s exact test. Boxplots comparing mRNA expression against protein detections were evaluated with an Independent-Samples Kruskal-Wallis Test. Cox analysis was performed to assess the independent prognostic significance of Claudin-6. No significant interactions were found between the included variables.

## Results

### High Claudin-6 associates with aggressive markers for EC, and is highly expressed in serous tumours

**Fig. 1:**
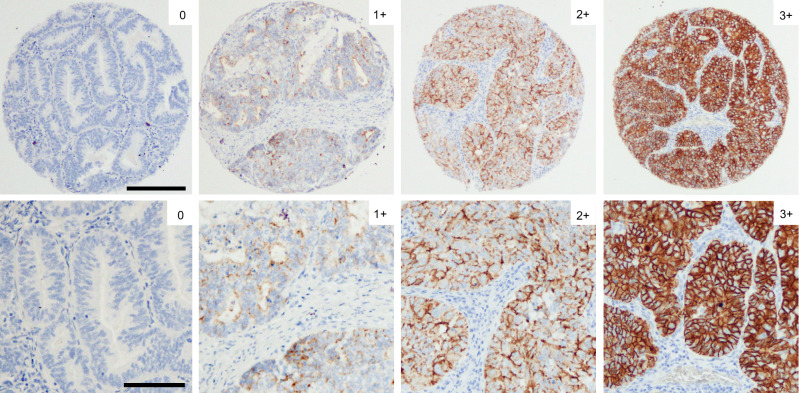
Differential expression of Claudin-6 in hysterectomy samples. Tissue microarrays were stained by immunohistochemistry and scored according to the Gastric criteria yielding the scores 0, 1 + , 2+ and 3 + . Scores reflect the level of membranous Claudin 6 expression levels. Scale bar top panel: 100 µm. Scale bar bottom panel: 50 µm.

Claudin-6 expression was evaluated in 1106 primary tumours using the Gastric score and revealed staining ranging from completely negative to strong staining (Fig. 1). No expression was seen in the stromal tissue. A notably high fraction of samples (82%) was completely negative for Claudin-6 and subsequently scored 0. Of the remaining 18% of the samples, 8% were scored 1 + , 6% were scored 2 + , and 4% of the samples were scored 3 + . A detailed description of distribution of scores 0, 1 + , 2 + , and 3+ across all tumours is given in Supplementary Table 1.

Samples were subsequently combined into two groups, where tumours with scores 0 and 1+ were classified as “low” and tumours with scores 2+ and 3+ as “high”. To identify patients that might be eligible for treatment with ADCs targeting Caludin-6, we explored expression patterns in the full patient population (Table 1). High Claudin-6 expression was significantly associated with clinicopathological features of aggressive disease. Patients with high Claudin-6 were significantly older (mean age 71 versus 66 years; *p* < 0.001) with non-endometrioid histology (*p* < 0.001) and high FIGO stage (*p *= 0.002). Few endometrioid tumours (4%) showed high Claudin-6. Within the NEEC subtypes, high Claudin-6 expression was most common in serous tumours (49%) and carcinosarcomas (22%), and less common in clear cell tumours (7%). None of the undifferentiated tumours showed high Claudin-6 expression. Among the few endometrioid tumours with high Claudin-6, a significant association was seen to higher grade (*p* < 0.001). High Claudin-6 expression significantly associated with the molecular subgroup of copy-number high (CNH) tumours, where 33% exhibited high Claudin-6. Gene expression data was available for a subset of patients.

**Table 1:** High Claudin-6 is associated with the clinicopathological factors of aggressive disease.

	Low n (%) (0 and 1 + )	High n (%) (2+ and 3 + )	*P*-value^a^
**Number of patients**	997 (90)	109 (10)	
**Age, median (range)**	66 (25-93)	71 (52-89)	**<0.001**^**b**^
**Histologic type**			**<0.001**^**c**^
Endometrioid	839 (96)	37 (4)	
Non-endometrioid	158 (69)	72(31)	
Serous	61 (51)	59 (49)	
Clear cell	38 (93)	3 (7)	
Carcinosarcoma	36 (78)	10 (22)	
Undifferentiated/other	23 (100)	0 (0)	
**Histologic grade**^**d**^			**<0.001**^**e**^
Grade 1	414 (99)	4 (1)	
Grade 2	286 (95)	15 (5)	
Grade 3	126 (88)	18 (12)	
**2009 FIGO stage**			**0.002**
I-II	852 (91)	81 (9)	
III-IV	145 (84)	28 (16)	
**Myometrial infiltration**			0.161
<50% or no infiltration	579 (91)	56 (9)	
≥50%	413 (89)	53 (11)	
**Molecular subtype**			**<0.001**^**e**^
POLE	76 (93)	6 (7)	
MMR-D	263 (97)	7 (3)	
CNL	393 (95)	21 (5)	
CNH	113 (67)	56 (33)	

*FIGO* International Federation of Gynecology and Obstetric.

Data missing on histological grade for 13 patients, myometrial infiltration for 5 patients, molecular subtype for 171 patients.

*POLE* DNA polymerase epsilon, *MMR-D* mismatch repair-deficient, *CNL* copy-number low, *CNH* copy-number high.

^a^Chi-square test.

^b^Kruskal Wallis Test.

^c^Non-endometrioid versus endometrioid.

^d^Endometrioid tumors only.

^e^Fisher exact test.

Significant p-values are given in bold.

*CLDN6* expression was significantly correlated with Claudin-6 (IHC) expression levels (Supplementary Fig. 1). High *CLDN6* mRNA levels and distinct gene expression patterns were identified for patients with high Claudin-6 protein expression (Supplementary Fig. 2). Gene sets linked to membrane transporter activity was enriched in patients with high Claudin-6 in GSEA analysis (Supplementary Table 2).

### Claudin-6 is an independent marker of poor survival in EC

**Fig. 2:**
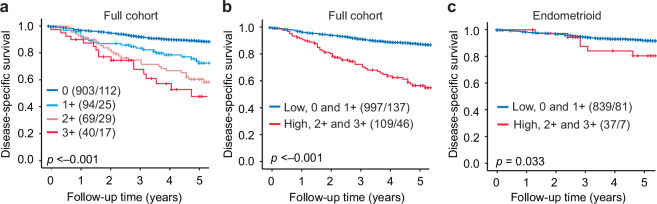
High Claudin-6 expression associates with poor prognosis. Kaplan–Meier plots illustrating disease-specific survival according to immunohistochemical Claudin-6 expression levels, assessed by the Gastric criteria (*n* = 1106). Survival outcomes for patients within each scoring category (0, +1, +2, and +3) **(a)** and for low (score 0 and +1) and high (score +2 and +3) expression groups in the full cohort **(b)** and in the endometrioid subgroup only **(c).** Numbers in brackets: total number of patients/number of events.

Implementing an ADC-target as a response marker in the clinic may improve prognostication if the selected marker has an additional independent prognostic value. To assess the prognostic value of Claudin-6, survival analyses were performed. In univariate analysis, Claudin-6 expression in four groups significantly correlated with DSS, ranging from a five-year DSS rate of 89% in patients with no expression and decreased to 72% with a score of 1 + , 59% with a score of 2 + , and 48% with a score of 3+ (*p* < 0.001, Fig. 2a). When comparing high versus low expression, high expression of Claudin-6 predicted a 5-year DSS rate of 55% compared to 87% for low expression (*p* < 0.001, Fig. 2b). High Claudin-6 associated with poor DSS also within the endometrioid subgroup (*p* = 0.033, Fig. 2c).

To explore whether Claudin-6 is an independent prognostic marker, multivariable Cox regression analysis including age and ESMO postoperative risk in three groups, was performed. High Claudin-6 expression independently predicted reduced disease-specific survival (HR 1.7, 95% CI 1.2–2.4, *p* = 0.002), indicating a 70% increased risk of disease-related death compared to low expression (Table 2). Within endometrioid tumours alone, Claudin-6 was not an independent prognostic marker.

**Table 2:** Claudin-6 expression is an independent postoperative prognostic marker in EC.

Variable	*N*	Univariable HR (95% CI)		*p*-value	Multivariable HR (95% CI)		*p*-value
**Age**	1093	1.06	(1.04–1.07)	***p ***< **0.001**	1.04	(1.02-1.05)	***p*** < **0.001**
**Postoperative ESMO risk group**							
Intermediate(ref)	235						
Low	424	0.2	(0.1–0.5)	***p*** < **0.001**	0.2	(0.1-0.6)	***p*** = **0.001**
High	434	6.2	(3.8–10.1)	***p*** < **0.001**	5.5	(3.4-9.1)	***p*** < **0.001**
**Claudin-6**							
Low (0-1) (ref)	984						
High (2-3)	109	4.0	(2.8–5.6)	***p*** < **0.001**	1.7	(1.2-2.4)	***p*** = **0.002**

Number of events: 183. Significant p-values are given in bold.

*HR* hazard ratio; *CI* confidence interval.

### Claudin-6 is highly expressed in metastatic EC lesions from serous tumours and carcinosarcomas

**Fig. 3:**
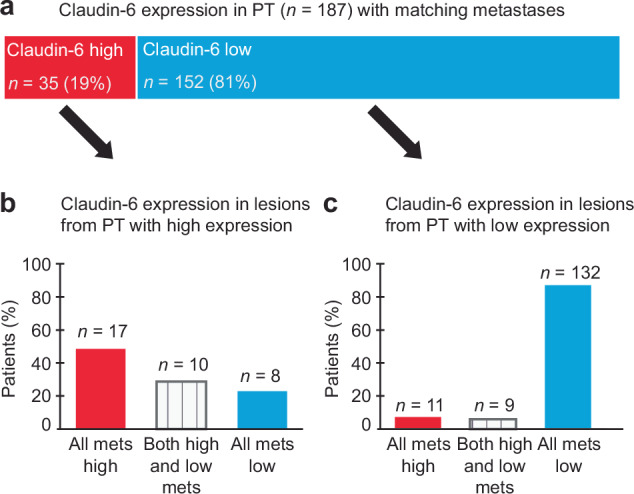
Claudin-6 is frequently expressed in metastatic lesions. Analysis of paired primary tumours (PT) and corresponding metastatic lesions (*n *= 187). Number of patients with high (2+ and 3 + ) and low (0 and 1 + ) Claudin-6 expression in PTs (**a**). Claudin-6 expression levels in metastatic lesions from patients with high (2+ and 3 + ) expression in PTs (**b**). Claudin-6 expression levels in metastatic lesions from patients with low (0 and 1 + ) expression in PTs (**c**).

Successful treatment of advanced EC relies on treatment response in the metastatic lesions. As EC is known to be a heterogeneous disease, protein expression in primary tumours may not represent expression in corresponding metastatic lesions. In the whole cohort of primary tumours, 10% (109 patients) had tumours with high expression of Claudin-6. In total, 187 patients had corresponding primary tumours and metastatic lesions available. Within this paired cohort, 19% (35 patients) had high Claudin-6 expression (Fig. 3a), reflecting the association of high Claudin-6 and aggressive disease. Among these, 77% (27 patients) retained high expression in either all (*n* = 17 patients) or some (*n* = 10 patients) of the metastases, while 23% (8 patients) lost expression and were categorized as low tumours in the metastatic setting (Fig. 3b). For the 81% (152 patients) with low Claudin-6 expression in their primary lesion, high expression in one or more metastatic lesion was observed in 13% (20 patients) while the remaining 87% (132 patients) retained low expression across corresponding lesions (Fig. 3c). In total, 25% (47 patients) had one or more metastatic lesions with high expression of Claudin-6.

**Fig. 4:**
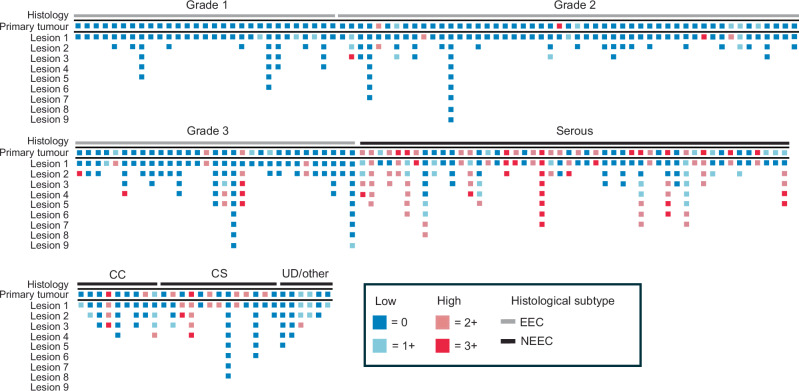
Claudin-6 expression is retained in metastatic lesions. Tile plot illustrating Claudin-6 expression in primary tumours and matched metastatic lesions. CC Clear Cell, CS Carcinosarcoma, EEC Endometrioid endometrial cancer, NEEC Non-endometrioid endometrial cancer.

A tile plot illustrates the expression patterns across EC subtypes (Fig. 4), with a higher prevalence of highly expressing primary tumours in NEEC subtypes compared to EECs. High Claudin-6 was very rarely observed in metastatic lesions from low grade endometrioid tumours and, similarly, few metastases from grade 3 EEC tumours were Caludin-6 high. A high fraction of metastatic lesions from both the serous tumours and the carcinosarcomas were Claudin-6 high, suggesting that targeting Caludin-6 with ADCs is highly relevant for treatment of metastatic disease in patients diagnosed with these subtypes.

## Discussion

Chemotherapy is the standard treatment for advanced and recurrent EC but is associated with development of resistance as well as severe and long-term side effects [22]. Antibody Drug Conjugates (ADCs) have emerged as a promising advancement, offering targeted delivery of chemotherapy with marked reduction in off-target effects [23]. Despite a rapid development of ADCs and increased number of approvals for clinical use, ENHERTU^®^, targeting HER2, is the only ADC that has reached approval for EC by the FDA, but is not yet approved by the EMA [24]. However, several ADCs may have efficacy in EC and a detailed mapping of expression of candidate targets is urgently needed. In the present study we explore Claudin-6 as a potential ADC target in EC. Our data show that Claudin-6 is highly expressed in aggressive EC tumours, independently predicts poor DSS, and, importantly, is frequently expressed in metastatic lesions from serous tumours and carcinosarcomas.

Claudin-6 is a membrane bound protein involved in cell-cell interactions and has been linked to prognosis in multiple cancers [10, 13, 14, 25, 26]. However, its role in tumour growth and metastasis is unclear and likely context dependent. In our population-based cohort of 1106 primary tumours, positive Claudin-6 expression (≥1+) was found in 18% of all primary lesions and high expression (≥2+) was found in 10% of primary tumours. We found highest Claudin-6 expression in tumours from patients with advanced FIGO stage and non-endometrioid tumour subtypes; particularly serous tumours and carcinosarcomas. In a previous study evaluating *CLDN6* mRNA expression in an EC cohort of 551 patients, 91% of serous tumours showed high *CLDN6* [14]. In our cohort, 68% and 49% of serous tumours showed ≥ 1+ and ≥2+ positive Claudin-6 protein expression, respectively. We also identified that high Claudin-6 expression was more prevalent in copy-number high tumours, also in line with the EC study evaluating *CLDN6* mRNA expression [14]. Interestingly, in gastric cancer, high *CLDN6* associates with *TP53* mutations and poor outcomes, and 97% of *CLDN6* high tumours are classified as chromosomal unstable molecular subtype [27]. High Claudin-6 expression associates with poor survival in bladder, cervical, endometrial and adrenocortical cancer [13–15]. However, high Claudin-6 expression has been linked to improved survival in non-small cell lung cancer, breast cancer, and specific subtypes of colorectal cancer [10–12]. A tumour-suppressive role of Claudin-6 has been functionally demonstrated in breast cancer cells, whereas an oncogenic role has been demonstrated for endometrial, hepatocellular and gastric cancer [28–32]. In our large, population-based EC cohort, high Claudin-6 associates with poor DSS. Importantly, after adjusting for age and ESMO postoperative risk, Claudin-6 remains an independent prognostic marker in a multivariable Cox regression analysis. Thus, in addition to an ADC target, Claudin-6 expression may aid in risk stratification of patients.

Claudin-6 has a strong potential as an ADC target across different tumour types due to positive expression in cancer tissues and no or minimal expression in normal tissues [33]. This observation has led to the initiation of Phase I clinical trials (NCT05103683, NCT05394675, NCT06932094) to evaluate the potential of targeting Claudin-6 with ADCs in solid tumours. However, it is still unclear which subtypes of endometrial tumours that are more likely to respond to treatment. We here identify a subgroup of EC patients (10%) that positively express Claudin-6 in primary tumours. This subgroup includes the most aggressive EC tumours where adjuvant platinum-based chemotherapy is the current main treatment. Unfortunately, about 50% of these patients show poor response and rechallenging with platinum-based chemotherapy has limited effect [34]. TORL-1-23 has demonstrated activity in platinum-resistant ovarian cancers that share biological traits with serous ECs [35]. This may suggest that platinum-resistant serous ECs also could benefit from treatment with TORL-1-23. Final data from this study, including from EC patients, will be highly interesting and will help inform future clinical trials with Claudin-6 targeting ADCs.

Successful treatment of metastatic EC depends on the response in metastatic lesions. High intra-tumour heterogeneity is common in EC and marker expression in primary tumours may not mirror marker expression in the corresponding metastatic lesion(s). To our knowledge, we here present the first study that evaluates Claudin-6 expression patterns between matched primary and metastatic lesions. A total of 77% of patients with high Claudin-6 expression in primary tumours maintained high expression in at least one corresponding metastasis. Of these, 48% showed positivity in all metastatic lesions, whereas 29% had one or more Claudin-6 positive metastatic lesions. For the latter subgroup, this heterogeneous expression may indicate that ADCs targeting Claudin-6 may not be sufficient to cure the disease but could reduce disease burden and prolong survival time. Overall, Claudin-6 is a valuable target for treatment of metastatic disease. Interestingly, in our cohort, Claudin-6 was enriched in tumours with metastatic potential. Specifically, in the metastasis cohort, high Claudin-6 expression was detected in 19% of primary tumours compared to only 10% in the population-based cohort. This pinpoints Claudin-6 as a potential driver of metastasis in EC. Claudin-6 is a critical component of tight junctions [8]. Although few functional studies have been performed to date, studies using EC cell line models suggest that Claudin-6 is involved in regulation of cell proliferation, migration and invasion [13, 31]. Specifically, knocking down Claudin-6 decreased proliferation and migration of Hec-1B cells and overexpression in Ishikawa cells had opposite effects. In Ishikawa xenografts, Claudin-6 overexpression enhanced tumour growth and invasion. Both studies also suggest a link between Claudin-6 and PI3K/AKT/mTOR and oestrogen signalling. However, these findings should be interpreted with caution due to methodological constraints. Future studies should focus on the use of advanced patient-derived EC model systems to map out pathway interactions and determine its role in tumour progression and metastasis. Claudin-6 is highly expressed in a subset of serous and carcinosarcomas and, regardless of its role as a driver of disease progression, Claudin-6 may serve as an efficient target for ADC treatment in EC.

Successful treatment of cancer patients using ADC-based strategies relies on specific interactions between the ADC and the antigen. In the present study, we use a commercially available antibody targeting Claudin-6 and identified that 5% of EC tumours have high Claudin-6. Although we cannot rule out any cross-reaction with other antigens, e.g. Claudin 9 [36], our gene expression data show a significant correlation between Claudin-6 protein and *CLDN6* mRNA expression and no correlation with other Claudin family members, indicating target specificity. Still, as for all biomarkers studies, target specificity needs to be validated and optimal staining conditions and scoring cutoffs should be determined before clinical implementation.

## Conclusion

Our study demonstrates Claudin-6 as an independent prognostic marker in EC. The association with poor survival and features of aggressive disease suggests that performing IHC for Claudin-6 expression would add prognostic information and aid clinical decision making, alongside identifying patients eligible for ADCs. The high expression of Claudin-6 in both primary and metastatic lesions of particularly aggressive endometrial tumours, highly review and appsupports further efforts to target Claudin-6 with ADCs in clinical trials.

## Supplementary information

Supplementary information

## Data Availability

All relevant data are included in figures, tables and supplementary material. Raw data are available upon reasonable request to the corresponding author ([Camilla.Krakstad@uib.no]), pending ethics review and approval.
