# Supplementary material for: High expression of the ADC target Claudin-6 associates with aggressive endometrial cancer and remains high in metastatic lesions
Source: BJC Rep. 2026 Apr 15;4:17. doi: 10.1038/s44276-026-00225-x (PMC13083938; doi:10.1038/s44276-026-00225-x)
Supplement: Supplementary file 1 — Supplementary information [file 44276_2026_225_MOESM1_ESM.pdf]

Supplementary Figures

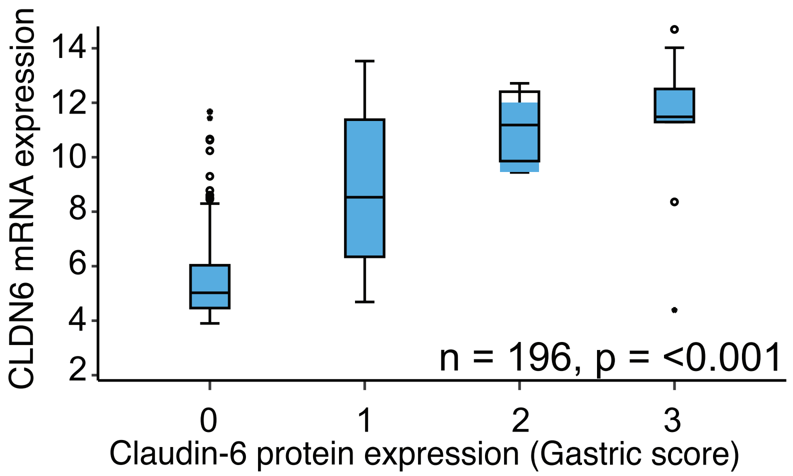

Supplementary Figure 1. CLDN6 expression levels in relation to Claudin-6 protein expression levels. Boxplot showing CLDN6 VST-transformed mRNA levels against Claudin-6 Gastric scores (0, 1+, 2+ and 3+) from immunohistochemically (IHC) stained tissue slides.

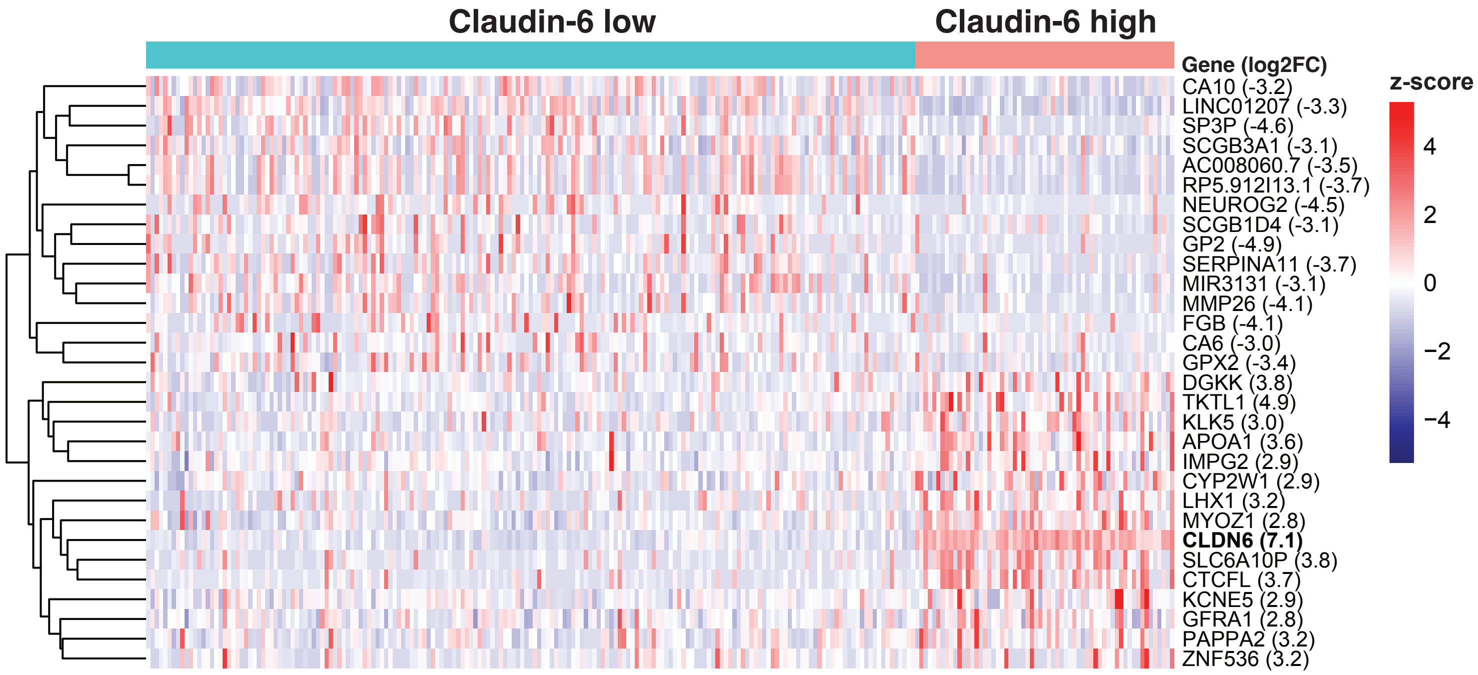

Supplementary Figure 2. Gene expression profiles in tumours with high versus low Claudin-6 expression. Heatmap showing expression patterns of the top 15 upregulated and top 15 downregulated genes (log2FC, FDR < 0.001) in tumours in the high (n = 61) versus low (n=181) Claudin-6 IHC expression groups. High mRNA expression is indicated in red and low mRNA expression in indicated in blue. Fold change for individual genes is given in brackets.

Supplementary tables

Supplementary Table 1. Clinicopathological characteristics in relation to Claudin-6 in four IHC expression groups

	Claudin-6, n (%)				
	**0**	**+1**	**+2**	**+3**	**p-value[a]**
**Number of patients**	903 (82)	94 (8)	69 (6)	40 (4)	
**Age, median (range)**	65 (25-93)	70 (46-93)	70 (52-89)	71 (59-87)	**<0.001[b]**
**Histologic type**					**<0.001[c]**
Endometrioid	781 (89)	58 (7)	25 (3)	12 (1)	
Non-endometrioid	122 (53)	36 (16)	44 (19)	28 (12)	
Serous	38 (32)	23 (19)	34 (28)	25 (21)	
Clear cell	36 (88)	2 (5)	1 (2)	2 (5)	
Carcinosarcoma	27 (59)	9 (20)	9 (20)	1 (2)	
Undifferentiated/other	21 (91)	2 (9)	0 (0)	0 (0)	
**Histologic grade[d]**					**<0.001[e]**
Grade 1	395 (94)	19 (4)	3 (1)	1 (0)	
Grade 2	264 (88)	22 (7)	8 (3)	7 (2)	
Grade 3	109 (75)	17 (12)	14 (10)	4 (3)	
**FIGO stage**					**0.011**
I-II	775 (83)	77 (8)	53 (6)	25 (2)	
III-IV	128 (72)	11 (10)	16 (9)	12 (7)	
**Myometrial infiltration**					0.568
<50% or no infiltration	525 (83)	54 (8)	36 (6)	20 (3)	
≥50%	375 (81)	38 (8)	33 (7)	20 (4)	
**Molecular subtype**					**<0.001[e]**
POLE	68 (83)	8 (10)	4 (5)	2 (2)	
MMR-D	252 (93)	11 (4)	6 (2)	1 (0)	
CNL	370 (89)	23 (6)	17 (4)	4 (1)	
CNH	79 (47)	34 (20)	35 (21)	21 (12)	

FIGO: International Federation of Gynecology and Obstetric
Data missing on histological grade for 13 patients, myometrial infiltration for 5 patients, molecular subtype for 171 patients.
POLE: DNA polymerase epsilon, MMR-D: mismatch repair-deficient, CNL: copy-number low, CNH: copy-number high.
[a] Chi-square test
[b]Kuskal Wallis test
[c] Endometrioid versus non-endometrioid
[d] Only within endometrioid tumors
[e]Fisher's exact test
[f]Monte Carlo significance test was used due to insufficient memory to perform Fisher`s Exact test

Supplementary Table 2. Upregulation of *CLDN6* is associated with calcium ion binding and transporter activity.

Rank	Gene Set Name	Size	FDR q-value
**1**	CALCIUM_ION_BINDING*	20	3.8E−06
**2**	MONOATOMIC_ION_TRANSMEMBRANE_TRANSPORTER_ACTIVITY*	20	6.94E−06
**3**	TRANSPORTER_ACTIVITY*	25	8.04E−06
**4**	INORGANIC_MOLECULAR_ENTITY_TRANSMEMBRANE_TRANSPORTER_ACTIVITY*	19	8.04E−06
**5**	GATED_CHANNEL_ACTIVITY*	13	8.04E−06
**6**	METAL_ION_TRANSMEMBRANE_TRANSPORTER_ACTIVITY*	15	8.04E−06
**7**	VOLTAGE_GATED_CHANNEL_ACTIVITY*	10	1.05E−05
**8**	KRAS.600.LUNG.BREAST_UP.V1_DN	12	1.2E−05
**9**	MONOATOMIC_CATION_TRANSMEMBRANE_TRANSPORTER_ACTIVITY*	16	1.06E−04
**10**	PASSIVE_TRANSMEMBRANE_TRANSPORTER_ACTIVITY*	14	1.37E−04

*Gene ontology: molecular function (GO:MF). 274 genes were included in the comparison.
